# Minocycline Down-Regulates Topical Mucosal Inflammation during the Application of Microbicide Candidates

**DOI:** 10.1371/journal.pone.0043211

**Published:** 2012-08-14

**Authors:** Liangzhu Li, Yinyin Ben, Zhaoqin Zhu, Weihua Li, Jianqing Xu, Xiaoyan Zhang

**Affiliations:** 1 Shanghai Public Health Clinical Center and Institutes of Biomedical Sciences, Fudan University, Shanghai, China; 2 Key Laboratory of Medical Molecular Virology, Institute of Medical Microbiology, Shanghai Medical College of Fudan University, Shanghai, China; 3 State Key Laboratory for Infectious Disease Prevention and Control, Chinese Center for Disease Control and Prevention, Beijing, China; 4 Shanghai Institute of Planned Parenthood Research, World Health Organization Collaborating Centre for Research in Human Reproduction, Shanghai, China; University of Central Florida College of Medicine, United States of America

## Abstract

An effective anti-human immunodeficiency virus-1 (HIV-1) microbicide should exert its action in the absence of causing aberrant activation of topical immunity that will increase the risk of HIV acquisition. In the present study, we demonstrated that the vaginal application of cellulose sulfate (CS) gel induced topical mucosal inflammatory responses; the addition of minocycline to CS gel could significantly attenuate the inflammation in a mice model. The combined gel of CS plus minocycline not only reduced the production of inflammatory cytokines in cervicovaginal lavages (CVLs), also down-regulated the activation of CD4+ T cells and the recruitment of other immune cells including HIV target cells into vaginal tissues. Furthermore, an *In vitro* HIV-1 pseudovirus infection inhibition assay showed that the combined gel decreased the infection efficacy of different subtypes of HIV-1 pseudoviruses compared with that of CS gel alone. These results implicate that minocycline could be integrated into microbicide formulation to suppress the aberrant activation of topical mucosal immunity and enhance the safety profile during the application of microbicides.

## Introduction

The prerequisite for microbicide application in the vaginal or rectal tract to prevent HIV-1 sexual transmission is that no aberrant activation of local immunity could be induced, since the mucosal topical inflammation will erode epithelial continuity and directly trigger HIV-1 replication [1]–[3]. In the first generation of HIV-1 microbicides, surfactant-based candidates such as nonoxynol-9 (N-9) nonspecifically disrupted phospholipid membranes of both host cells and microorganisms, induced inflammation and finally facilitated entry of HIV-1 [4], [5]. Other polyanionic-based candidates such as cellulose sulfate (CS) showed better tolerance on vaginal or rectal epithelium and flora [6], however, they still triggered nuclear factor κB activation and induced secretion of inflammatory cytokines [3], [7]. The second generation of microbicide candidates consisted mainly of antiretroviral agents, entry inhibitors and mAbs. These provided much more specific anti-HIV activity and better tolerance within vaginal or rectal environments. However, the concerns of activating topical mucosal immunity still exist because a considerable number of these microbicides are peptides and antibodies [8], and might function as exogenous antigens for the mucosal immune system. Therefore, it is necessary to develop immunomodulators combined with the potential microbicide candidates to blunt aberrant activation of mucosal immunity and assure the safety of topical application of microbicides.

Recently, it was reported that minocycline, a second-generation tetracycline derivative, could attenuate HIV-1 infection and replication by suppressing the activation of CD4+ T cells [9]. Thus, minocycline was considered as a new class of “anti-cellular anti-HIV drugs” and was proposed to be an adjunctive treatment to the highly active antiretroviral therapy (HAART) [10]. However, the effect of minocycline on vaginal topical mucosal immunity and its resistance to aberrant activation of local immunity remained unclear.

In the present study, we screened the concentration range of minocycline which could be safely used in the genital tract without destroying normal flora and epithelial layers. Then, using these parameters, minocycline was evaluated with microbicide candidates for its mucosal immunomodulatory ability. The results suggested that minocycline has the potential to be combined with microbicides to reduce the risk of HIV-1 infection by suppressing aberrant activation of topical mucosal immunity.

## Methods

### Minocycline supply and formulation

Capsules containing 50 mg minocycline hydrochloride (Wyeth Pharmaceuticals) were used for the experiment described. The granuliform drug was removed from the capsule, then crushed to a powder and dissolved in 10 ml sterile phosphate-buffered saline (PBS) (pH 7.4 for cell experiment and pH 5.0 for animal experiment) at 37°C; centrifuged in 200×g for 5 min. Supernatant was then sterile-filtered to remove a small amount of insoluble material (medicine excipients). The actual concentration and purity of minocycline in the filtered supernatant was determined by high performance liquid chromatography (HPLC) (Figure S1) as described below, and then adjusted to final indicated concentrations. For vaginal application to mice, 1.5% hydroxyethyl cellulose (HEC) (catalog#434973, Sigma-Aldrich, Saint-Louis, MO, USA) or 60 mg/ml cellulose sulfate (catalog#SLC1798, Sciencelab.com, Inc., Houston, Texas) were slowly added into the minocycline solution or PBS (PH 5.0), while maintaining rapid stirring until a gel was formed. The formulation gels were then vaginally applied to a mice model as described below. 4% nonoxynol-9 (N-9) (catalog# SLN1945, Sciencelab.com, Inc,) formulated by 1.5% HEC was also used as a positive control in histopathological examinations.

### HPLC assay

An Agilent 1100 HPLC instrument (Agilent Technologies) was used to determine minocycline concentration, with a Diamonsil C18 column (5 μm, 4.6×150 mm). The mobile phase consisted of acetonitrile∶water∶trifluorocetic acid (15∶85∶0.1,v:v) with a flow rate of 1.0 ml/min, and a detecting wavelength of 350 nm.

### The mice model

All animal experiments were reviewed and approved by the Institutional Animal Care and Use Committee (IACUC) of Shanghai Public Health Clinical Center. Six to eight week-old pathogen-free outbred BALB/c female mice were purchased from Shanghai SLAC Laboratory Animal Co., Ltd (Shanghai, China) and housed for at least one week before the experiments were conducted. Mice were injected subcutaneously with 2 mg of medroxyprogesterone acetate (Catalog#M1629, Sigma- Aldrich) five days before treatment, to hormonally synchronize mice [11]. Forty microliters of gel was delivered intra-vaginally twice daily for three consecutive days. The interval of two applications was 12 hours, with cervicovaginal lavages (CVLs) collected by washing with 200 μl of sterile PBS (pH 7.4) next morning (after another 12 hours), prior to that day's gel application [12]. Twelve hours after the last treatment, 200 μl CVLs samples were collected, of which 100 μl was used for vaginal flora detection immediately. Mice were then humanely euthanized, with the vaginal tissues being recovered for preparation of mononuclear cells (MNCs) or for histological examination as described below. The CVLs (except the part of last collection which was used for vaginal flora detection) were treated with 10× protease inhibitor (Complete Protease Inhibitor Cocktail; Roche Applied Science, Indiana, USA) and centrifuged at 200×g for 10 min at 4°C. The supernatants were collected and stored at −80°C for cytokine quantification.

### Vaginal flora examination

50 μl CVLs from the last collection were centrifuged at 8000×g for 2 min, the supernatants were discarded and the precipitants were used for vaginal smear. All slides were gram stained and bacteria on smears were counted in high power fields using microscopy with a 1000× magnification. The rest of the CVLs were diluted by 1∶10 and 50 μl diluent was inoculated on China blue agar plates (catalog# DJPM015, DingJie Bio-technologies Co., Ltd., Shanghai, China), blood agar plates (catalog# DJPM004, DingJie) and lactobacillus MRS agar plates (catalog# 288210, BD Biosciences). The plates were incubated anaerobically at 37°C in 5–10% CO_2_ for 48 hours, and then bacterial plaques with different morphological characteristics were picked out and gas chromatography-fatty acid methyl ester (GC-FAME) analyses were performed, as previously described [13] for identification of the bacterial isolates. Numbers of colony-forming units (CFU) of different bacterial strains on the plates were counted.

For *in vitro* test of minimum inhibitory concentration (MIC) of minocycline against vaginal bacteria, different isolates picked from the plates were amplified by using Mueller-hinton (MH) broth (catalog# CM0405, oxoid) at 37°C in 5–10% CO_2_ for 48 hours (O.D. 1.0). This was then diluted 1000-fold with fresh MH broth media to act as a test organism suspension. A 96-well sterile microtitre tray was labeled with the appropriate minocycline dilutions (five-fold gradient dilution from 100 μg/ml to 0.032 μg/ml). 10 μl of each antibiotic dilution was added to one row of wells for each bacteria isolate. 240 μl of test organism suspension was dispensed into the row of wells, include inoculated and uninoculated wells of antibiotic-free broth. This was covered with a lid and sealing tape and incubated at 37°C in 5–10% CO_2_ for 48 hours. MIC endpoint was read as the lowest concentration of antibiotic at which there is no visible growth.

### Histopathological examination

Formalin-fixed excised vaginal tissues were embedded in paraffin, and transversely sectioned with a microtome. The slides were stained with hematoxylin-eosin and subjected to a blind evaluation for epithelial cell disruption and inflammatory responses.

### Analysis of cytokines from CVLs

Analysis of cytokines from CVLs was conducted using a mouse cytokine cytometric bead array (CBA) kit (Catalog#560485, BD Biosciences) and was analyzed on a FACAria flow cytometer (BD Biosciences). Standard curves were generated for each cytokine from a range of 0–5000 pg/ml, and 50 μl of each sample was used for analysis of cytokines by CBA assay. According to the manufacturer's instruction, the detection threshold for these assays ranges from 0.03–16.80 pg/ml, depending on the analyte.

### Preparation of genital tract MNCs

For isolation of mononuclear cells (MNCs), each uterus and the genital tract, including the cervix, was removed and cut into 0.5 cm pieces which were then rinsed with Ca-Mg-free Hanks' balanced salt solution (HBSS) (tissues from 5 mice in same group were pooled). Then, the tissues were incubated in a mixture of 5 mM EDTA and Ca-Mg-free HBSS at 37°C for 15 min with gentle stirring. The tissues were washed with PBS and cut with scissors (about 1–3 mm slices). The tissue was then incubated with RPMI 1640 containing 10% bovine calf serum, antibiotics (penicillin and streptomycin) and 0.5 mg/ml collagenase type V (Catalog#C9263, Sigma-Aldrich). This was incubated at 37°C with stirring for 30 min, three times. The isolated cells were pooled together and separated on a 40/75% discontinuous Percoll gradient (Catalog#17-0891-09, GE Healthcare Life Sciences, Chalfont St Giles, UK), centrifuged at 600×g at 25°C for 20 min. Cell layers for MNCs were recovered and suspended in complete RPMI 1640 at 4°C until use.

### Immunofluorescence staining and FACS analysis

The isolated MNCs from mice tissues were stained with surface antibodies conjugated to different fluorochromesfor 30 min at 4°C, including anti-CD3-Pacific blue, anti-CD4-APC/Cy7, anti-CD8-FITC, anti-CD11c-PE/Cy7, anti-CD335(NKp46)-PE, anti-CD14-PerCP/Cy5.5, and anti-TCRγδ-APC for cell subset phenotyping; anti-CD25-PerCP/Cy5.5, anti-CD69-PE, and anti-CD71-PE/Cy7 were used as activation markers. All antibodies were purchased from Biolegend Inc. (San Diego, CA, USA). Analysis was conducted on ≧10,000 gated viable lymphocytes based on FMO (fluorescence minus one) controls.

### HIV-1 pseudovirus infection inhibition assay

We used TZM-bl cells for the HIV-1 pseudovirus infection inhibition assay. Approximately 1×10^4^ cells/well was plated on a 96-well plate in Dulbecco's modified Eagle's medium containing 10% fetal bovine serum and penicillin-streptomycin. Each reagent in combination (0.3 μg/ml CS combined with 50 μg/ml minocycline) or alone (50 μg/ml minocycline or 0.3 μg/ml CS)) was subsequently added, and cells were treated for 2-8 h. Four kinds of HIV-1 Env-pseudotyped viruses, which were produced in 293T cells by co-transfection with four HIV-1 clades (subtype B-SVPB16, subtype C-SVPC12, CRF07_BC-32-72, CRF01_AE-SH188.6) Env expression plasmid and HIV-1 backbone plasmid expressing the entire HIV-1 genome except Env, pNL4-3Δenv [14], were then added respectively by 100TCID_50_ per well. Each reagent for each pseudotyped virus was performed in triplicate, and then incubated at 37°C with 5% CO_2_. After 48 h at 37°C, the cells were lysed in the presence of Bright-Glo (Catalog# E2620, Promega Corporation, Madison, USA), and relative luminescence was recorded by using a Victor3 luminometer (PerkinElmer, Inc. Massachusetts, USA).

### Statistical analysis

Data are presented as the mean ± standard deviation (SD). Statistical significance between different groups was calculated with one way Analysis of Variance (ANOVA) and Tukey's post-hoc tests using GraphPad Prism version 5.0 software (San Diego, USA). Values of *p*<0.05 were considered as statistically significant.

## Results

### Investigation of the concentration range for rational minocycline administration as an immunomodulator within the vaginal microenvironment

**Figure 1:**
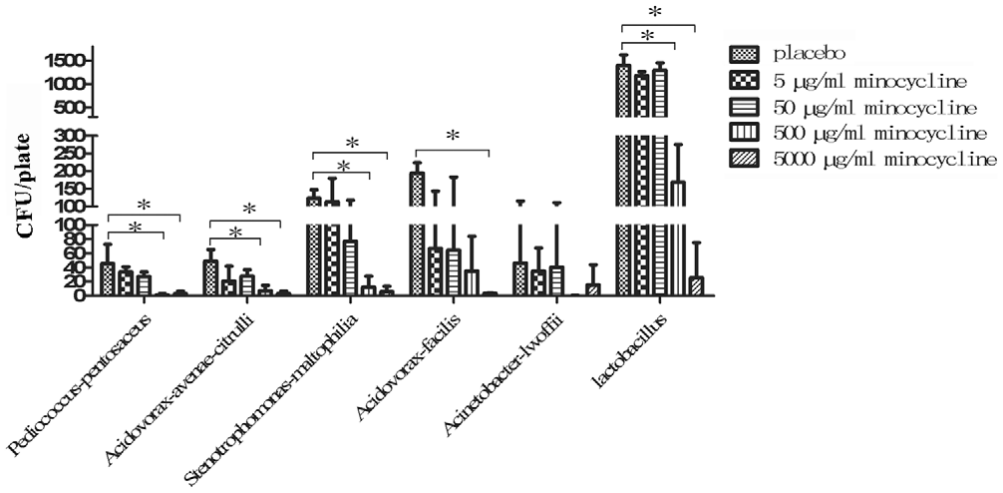
Effect of different concentrations of minocycline gel on the growth of vaginal microflora. X-axis indicated different bacteria isolates, Y-axis indicated the numbers of bacteria CFU on one agar plate. All bacteria strains were isolated from mice vagina and identified by GC-FAME analyses for their species notation with an instrument produced by Agilent Tech. Data represented as means ± SD from 4 mice per group. ANOVA and Tukey's post-hoc tests were performed among different groups. *: *p*<0.05.

To determine the concentration range of minocycline which is tolerant within the vaginal microenvironment, minocycline formulated at different concentrations together with HEC-gel were applied in the vagina of mice for three consecutive days, with CVLs collected for microflora growing assays. As shown by the vaginal smear test (Table 1 and Figure S2), nearly 100% of bacteriostatic efficacy was observed when minocycline was applied at a concentration of 500 μg/ml and 5000 μg/ml, with little influence on vaginal microorganisms seen when the concentration of minocycline was decreased to 50 μg/ml and 5 μg/ml. Via more careful analysis of agar plates isolation, GC-FAME identification and CFU counting, six main bacterial species in vaginal CVLs of mice were identified (*Pediococcus-pentosaceus, Acidovorax-avenae-citrulli, Stenotrophomonas-maltophilia, Acidovorax-facilis, Acinetobacter-lwoffii* and *lactobacillus*). With the exception of *Acinetobacter-lwoffii*, the growth of the other five isolates were all significantly inhibited by 5000 μg/ml and 500 μg/ml minocycline (except *Acidovorax-facilis*) (*p*<0.05), compared to a placebo control, while 50 μg/ml and 5 μg/ml minocycline had almost no significant effect (*p*>0.05) on the growth of all bacterial isolates (Figure 1). Therefore, 50 μg/ml was likely to be the safe concentration threshold for minocycline in HEC gel to be used on vaginal mucosa without destroying normal microflora.

**Table 1:** Bacteria counting on vaginal CVLs smear.

Group*	Mouse No.	Numbers of bacteria #				
		+	++	+++	++++	+++++
A	A1					√
	A2					√
	A3					√
	A4					√
B	B1					√
	B2					√
	B3					√
	B4					√
C	C1					√
	C2				√	
	C3					√
	C4					√
D	D1		√			
	D2		√			
	D3		√			
	D4		√			
E	E1	√				
	E2	√				
	E3	√				
	E4	√				

# Total number of bacteria in 10 random fields of microscope per slide at the magnification of 1000×. +: 0–9; ++:100–199; +++: 200–499; ++++: 500–1999; +++++: >2000.

* A: placebo; B: 5 μg/ml minocycline; C: 50 μg/ml minocycline; D: 500 μg/ml minocycline; E: 5000 μg/ml minocycline.

However, such safe concentrations *in vivo* were much higher than the *in vitro* MIC of minocycline, which ranged from 0.16 μg/ml to 0.8 μg/ml (Table S1). Previous work of J. D. Oriel, *et al.* [15] might give a good explanation for such phenomenon. Their work demonstrated that despite inhibition of the growth of yeasts shown by minocycline *in vitro*, there is no evidence of any significant inhibition on the growth of vaginal yeast flora *in vivo*, suggesting that there were too many unknown causes *in vivo* which would influence the effect of minocycline. Besides some unknown biological factors, the characteristics of gel formulation, such as its drug release rate, was likely to be another important reason resulting in divergence. This should be carefully studied in future research.

**Figure 2:**
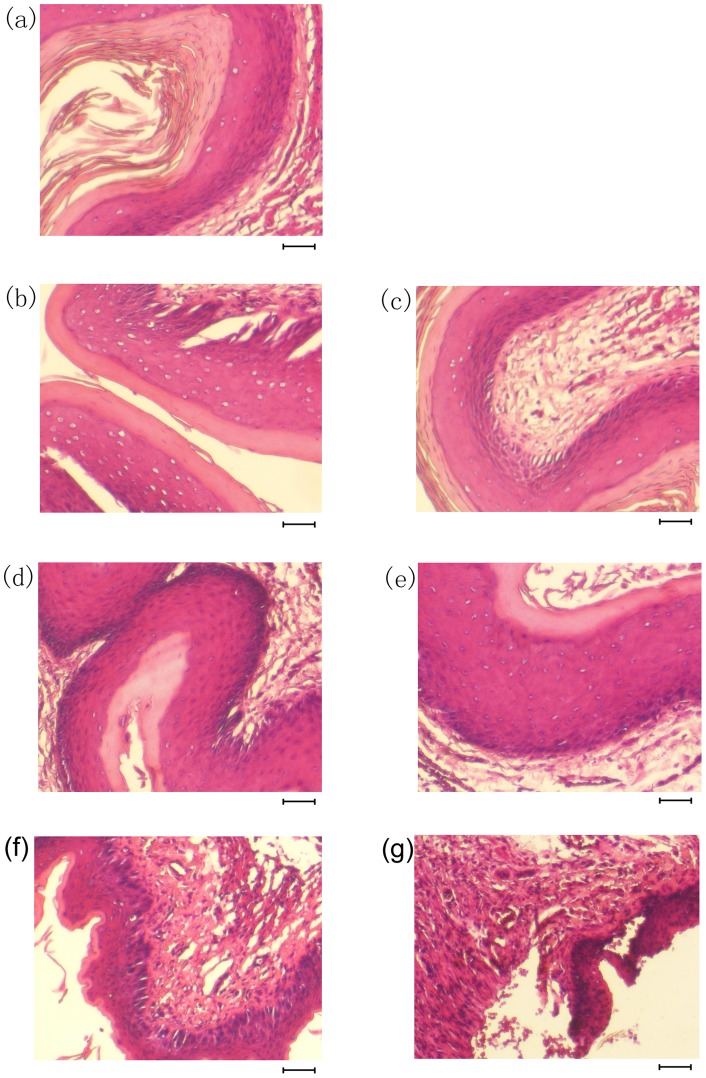
Epithelial integrity after intravaginal application of minocycline formulated with HEC gel at different concentrations. Vagina tissue sections were prepared and stained with hematoxylin-eosin. (a) represented the vagina tissue section from placebo (HEC gel) treated group, (b), (c), (d) and (e) represented the vagina tissue section from groups treated with 5 μg/ml, 50 μg/ml, 500 μg/ml and 5000 μg/ml minocycline gels respectively. 60 mg/ml CS (f) and 4% N-9 (g) had been shown to cause the disruption of epithelial integrity and were used as controls. Scale bars, 50 μm.

The histopathological examination of cervicovaginal tissues was also performed on day 3, following the intravaginal application of minocycline. No histopathological damage on vaginal epithelium was observed, regardless of the concentration of minocycline used (Figure 2). The results suggested that minocycline at 50 μg/ml should not disrupt the integrity of epithelial barriers, nor the normal flora within the vaginal microenvironment.

### Minocycline counteracted the effect of CS on upregulating secretion of inflammatory cytokine in CVLs

**Figure 3:**
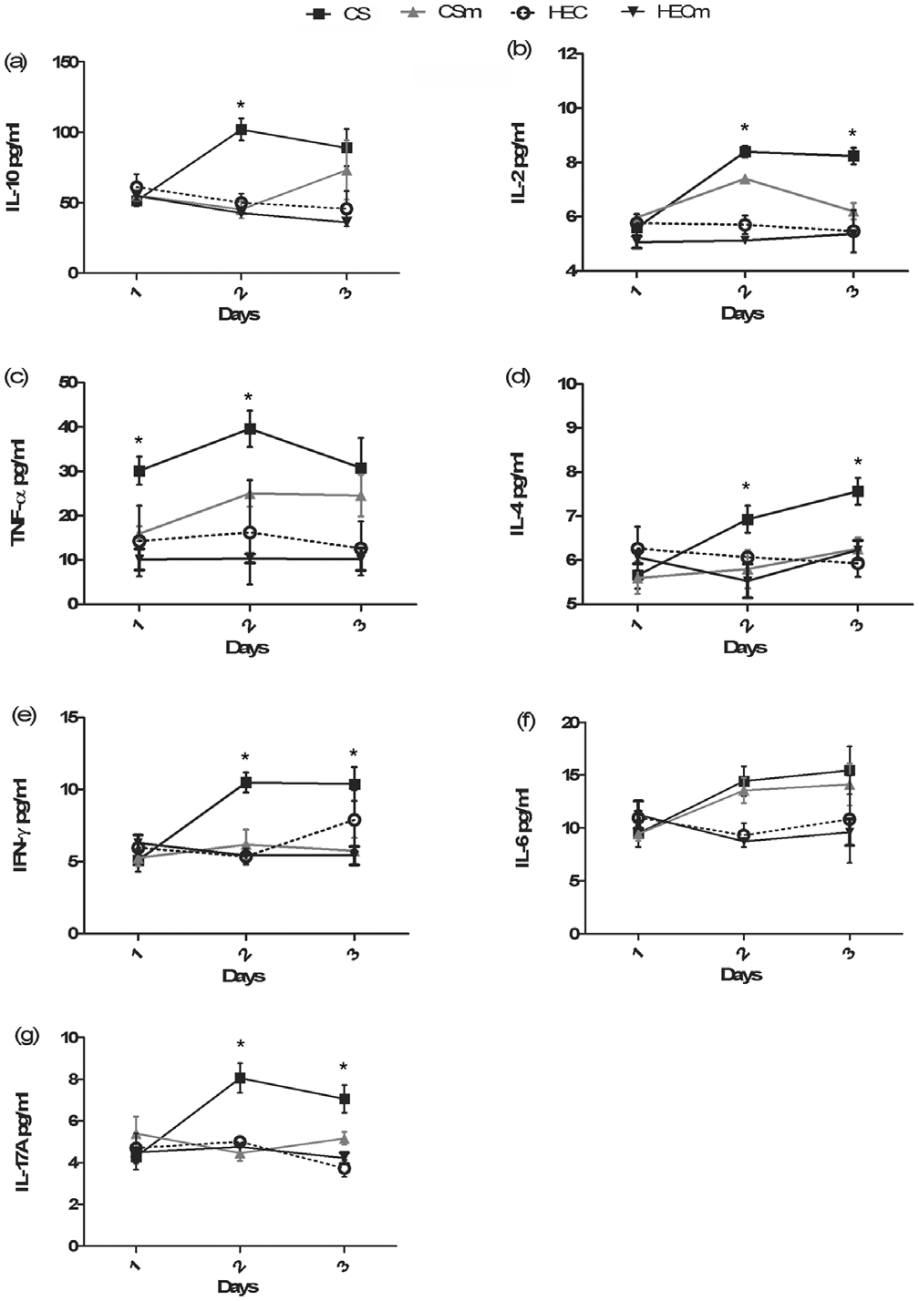
Dynamic profiles of different inflammatory cytokines after intravaginal application of different gel formulations. The inflammatory cytokines ((a) IL-10, (b) IL-2, (c) TNF-α, (d) IL-4, (e) IFN-γ, (f) IL-6, (g) IL-17A) for each mouse were quantified by CBA assay. The X-axis indicates the days after the administration of gels. Y-axis indicates the production of corresponding inflammatory cytokines. Data were represented as means ± SD from triplicate samples of CVLs pooled from 5 mice. In the graph, CS indicates gel containing 60 mg/ml CS alone; CSm indicates combined gel containing both 60 mg/ml CS and 50 μg/ml minocycline; HEC indicates a HEC placebo gel and HECm indicates HEC gel containing 50 μg/ml minocycline. ANOVA and Tukey's post-hoc tests were performed among different groups. The significant difference (*p*<0.05) between CS and CSm were indicated with * at certain time points.

We next investigated the potential of minocycline on pro-inflammatory cytokine secretion, which was considered as one of the biomarkers of safety evaluation for topical microbicide products [1], [2], [12], [16]. In present study, mice were divided into four groups and treated with (1) 60 mg/ml CS, (2)combination gel containing 60 mg/ml CS and 50 μg/ml minocycline, (3) 1.5% HEC placebo gel, and (4) 1.5% HEC gel containing 50 μg/ml minocycline, respectively, for three consecutive days. The dynamic productions of cytokines including interleukine-2 (IL-2), IL-4, IL-6, IL-17A, interferon-γ (IFN-γ), tumor necrosis factor-α (TNF-α), and IL-10 in CVLs were monitored daily by CBA as described above. As Figure 3 showed, all of the detected cytokines were significantly upregulated within mice treated with CS gel alone after 24 hours, peaking on day 2 or day 3 post treatment (*p*<0.05, compared with HEC placebo). In contrast, the production of cytokines in mice treated with CS gel containing minocycline were attenuated and most time points had statistically significant differences compared to CS gel alone (*p*<0.05). No prominent peak production of inflammatory cytokines was observed (except IL-2 and IL-6) compared to that in mice treated with HEC placebo gel. This data demonstrated that the combined application of minocycline with CS suppressed vaginal inflammation caused by CS.

### Minocycline attenuated recruitment and activation of immune cells caused by CS in vagina tissues

**Figure 4:**
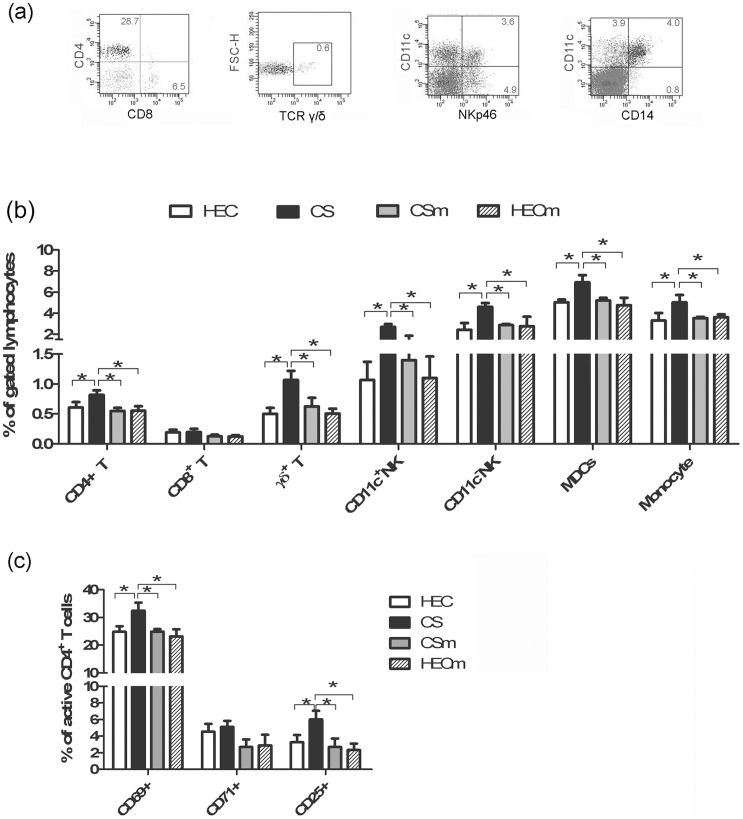
Effects of different gels on the recruitment and the activation of lymphocytes in vagina tissues. (a) was a representative for gating different cell subsets; the alteration of proportions of different cell subsets after the administration of different gels were summarized in graph (b), and alteration of activation markers expressed on CD4+ T cells in vaginal tissues were summarized in graph (c). Data were represented as means ± SD from triplicate cultures of cells pooled from 5 mice. ANOVA and Tukey's post-hoc tests were performed among different groups. *: *p*<0.05. In the graph, CS indicates gel containing 60 mg/ml CS alone; CSm indicates combined gel containing both 60 mg/ml CS and 50 μg/ml minocycline; HEC indicates a HEC placebo gel and HECm indicates HEC gel containing 50 μg/ml minocycline.

The mice described above were also used to determine the effect of minocycline on activation of CD4+ T cells and recruitment of local immune cells related to HIV-1 infection including T cells, NK cells, monocytes and dendritic cells (DCs) in vaginal tissues. As exemplified in Figure 4a, the following defining markers were used to define different cells and subsets: CD3/CD4/CD8 for αβ T cell subsets, γδ T cell TCR specific antibodies for γδ T cells, NKp46 receptor for NK cells, and CD14 and CD11c for monocytes (CD14+) and DCs (CD11c+CD14-). The data showed that application of 60 mg/ml CS resulted in recruitment of all examined immune cells at the topical vaginal sites (*p*<0.05), while combined CS gel containing 50 μg/ml minocycline crippled the recruitment of immune cells to the level of that in mice treated with HEC placebo gel or minocycline gel alone (Figure 4b). The change of cellular marker activation (CD25, CD69) and proliferation (CD71) for CD4+ T cells, typical target cells of HIV-1, were also investigated. As shown in Figure 4c, the application of CS caused the up-regulation of CD25 and CD69 in CD4+ T cells, while similar phenomena was not observed on cells treated with combined gel containing minocycline. This further corroborated the observation on recruitment. No up-regulation of CD71 was observed for CD4+ T cells, suggesting the proliferation was not initiated at this early stage. Altogether, these data demonstrated that minocycline could attenuate the recruitment of local immune cells and down-regulate the activation of T lymphocytes induced by CS within vaginal tissues.

### Minocycline provided an adjunctive effect to microbicide on prevention of HIV-1 infection

**Figure 5:**
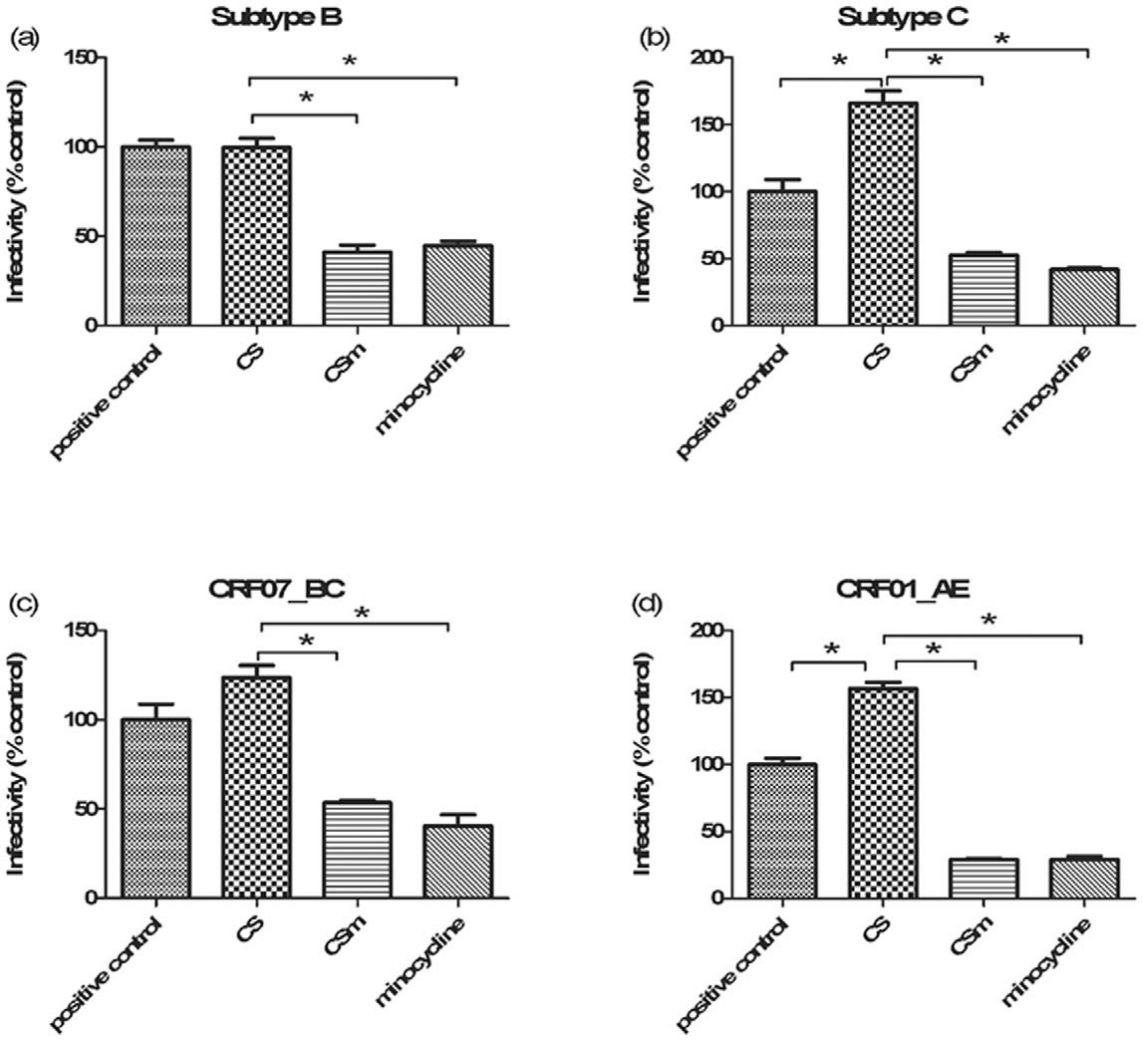
Effects of different gels on inhibition of HIV-1 pseudovirus infection *in vitro*. Four different subtypes of HIV-1 pseudovirus, including (a) B subtype, (b) C subtype, (c) CRF07_BC and (d) CRF01_AE, were employed in the experiment. Data were represented as means ± SD from triplicate experiment results. ANOVA and Tukey's post-hoc tests were performed among different groups, *: *p*<0.05. In the graph, untreated TZM-bl cells was used as a positive control, CS indicated 0.3 μg/ml CS treated cell group; CSm indicated a combined gel treated cell group containing both 0.3 μg/ml CS and 50 μg/ml minocycline and minocycline indicated 50 μg/ml minocycline treated cell group.

To further determine whether minocycline has an adjunctive effect to microbicides on prevention of HIV-1 infection, HIV-1 inhibition assays were performed with combined formulation containing 0.3 μg/ml CS and 50 μg/ml minocycline, 50 μg/ml minocycline alone or 0.3 μg/ml CS alone against different subtypes of HIV-1 pseudovirus (B, C, CRF07_BC, CRF01_AE). Consistent with the previous report [3], we confirmed that CS enhanced the infection rate of HIV; in contrast, both the combined CS gel containing minocycline and minocycline alone decreased the HIV-1 pseudovirus infection greatly (Figure 5), suggesting minocycline could suppress infection of HIV and counteract the effect of promoting infection of CS by alteration of cellular environment.

## Discussion

Mucosal topical application of the microbicide within the female genital tract was proven to effectively protect from HIV-1 and other sexually transmitted infections [17]. However, some microbicide candidates, such as CS, were halted in Phase III clinical trial, because this candidate increased the acquisition of HIV-1, which may be a result of the triggered aberrant activation of topical mucosal immunity by CS [3], [8], [18]. Therefore, it is an urgent need to explore new microbicide formulations which will be beneficial to microbicidal function and balance the topical mucosal immunity [19].

There is growing evidences that certain kinds of antibiotics (such as macrolides and tetracyclines) exert beneficial effects, not only by killing or inhibiting the growth of bacterial pathogens, but also by anti-inflammatory response [20]. These types of antibiotics tend to accumulate in inflammatory cells, down-regulate the expression of adhesion molecules and chemotactic factors on leukocytes, and significantly reduce the number of immune cells and the ratio of CD4+/CD8+ T lymphocytes [21]–[22]. Due to these immunomodulatory properties, these antibiotics have been widely used in clinical trials to treat immune dysfunction diseases [23].

Minocycline, a broad spectrum tetracycline antibiotic, also has significant tissue penetration and tends to accumulate in leukocytes for its lipophilicity [9], [24] It was studied for its immunomodulatory functions, including neuroprotective effects and anti-inflammatory activity for patients with rheumatoid arthritis, multiple sclerosis, and stroke in human trials [25]–[27]. Recently, it was reported that minocycline decreased the risk of HIV infection by inhibiting the transcription factors nuclear factor κB and activator protein 1, and resulted in alteration of T cell activation and cytokine secretion [9]. In the present study, we investigated the potential of minocycline as an immunomodulatory component in microbicide formulation. Through the mice model, it was demonstrated that minocycline at a concentration of ≤50 μg/ml neither disrupted contiguity of epithelium, nor destroyed normal vaginal flora. Inflammatory inducer CS, a failed microbicide candidate, was disarmed when co-applied with 50 μg/ml minocycline within the mouse vagina. In other words, the aberrant activation of topical mucosal immunity induced by CS, such as secretion of inflammatory cytokines, activation of T cells, and the recruitment of additional immune cells into vaginal mucosa, were all suppressed by minocycline. Furthermore, the anti-inflammatory effect of minocycline contributed to significant inhibition of HIV-1 infection caused by CS across a wide spectrum of subtypes (B, C, CRF07_BC and CRF01_AE). This study suggests minocycline has the potential to enhance the efficacy of a microbicide candidate via its immunomodulatory properties. In addition, minocycline is inexpensive and approved by the US Food and Drug Administration [10], and was proven to have low incidence of toxicity with long-term administration [9], [28]. All of these advantages make minocycline a promising immunomodulator that can be used to optimize the microbicide formulation and decrease the risk of HIV transmission.

There were some side effects caused by oral administration of minocycline, such as upset stomach, diarrhea, dizziness, unsteadiness and vomiting [29]–[31], therefore, monitoring the plasma concentrations of minocycline should be done after intravaginal application in future non-human primate experiments before enter into clinical trial. Effects of long-term intravaginal application of minocycline on mucosal immunity should also be investigated.

## Supporting Information

Figure S1 **Purity and concentration of minocycline monitored by HPLC.** The retention time was 5.75 min for minocycline. Purity of minocycline in capsule was 95%, and the concentration of minocycline in the filtered supernatant was 6 mg/ml.
(TIF)

Figure S2 **Bacteriostatic activity at different concentrations of minocycline gels.** Vaginal smears were prepared from vagina CVL precipitants, and then gram stained and examined under microscopy at a 400× magnification. (a) represented the vagina smear from placebo treated group, (b), (c), (d) and (e) represented vagina smears from groups treated with 5 μg/ml, 50 μg/ml, 500 μg/ml and 5000 μg/ml minocycline gels respectively. Scale bar: 20 μm.
(TIF)

Table S1 **MIC of minocycline against bacteria isolates.** The experiment was repeated for three times and data were represented as means ± SD. All bacteria strains were isolated from mice vagina and identified by GC-FAME analyses for their species notation with an instrument produced by Agilent Tech.
(DOC)
