# Supplementary figures and images for: Minocycline Down-Regulates Topical Mucosal Inflammation during the Application of Microbicide Candidates
Source: PLoS One. 2012 Aug 14;7(8):e43211. doi: 10.1371/journal.pone.0043211 (PMC3419165; doi:10.1371/journal.pone.0043211)

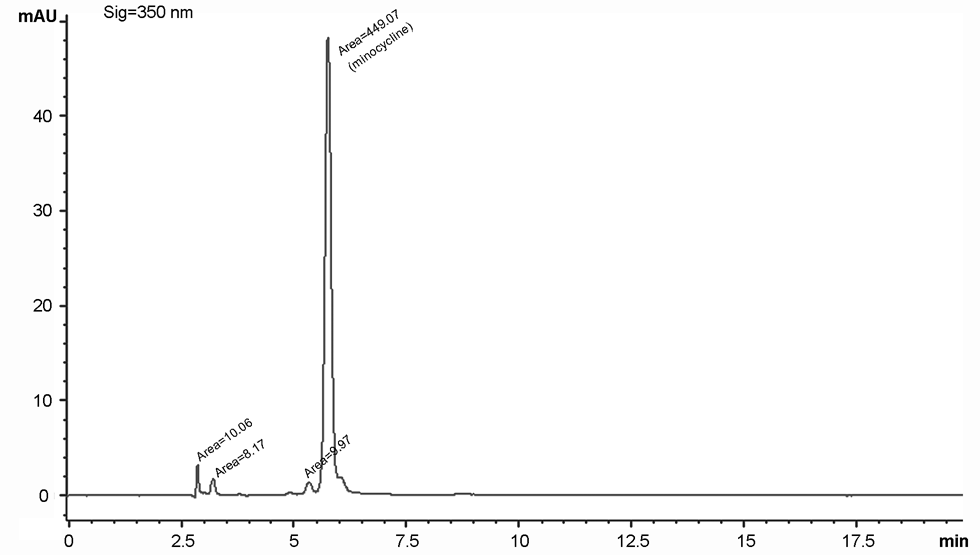

Supplement: Figure S1 — Purity and concentration of minocycline monitored by HPLC. The retention time was 5.75 min for minocycline. Purity of minocycline in capsule was 95%, and the concentration of minocycline in the filtered supernatant was 6 mg/ml. (TIF) [file pone.0043211.s001.tif]

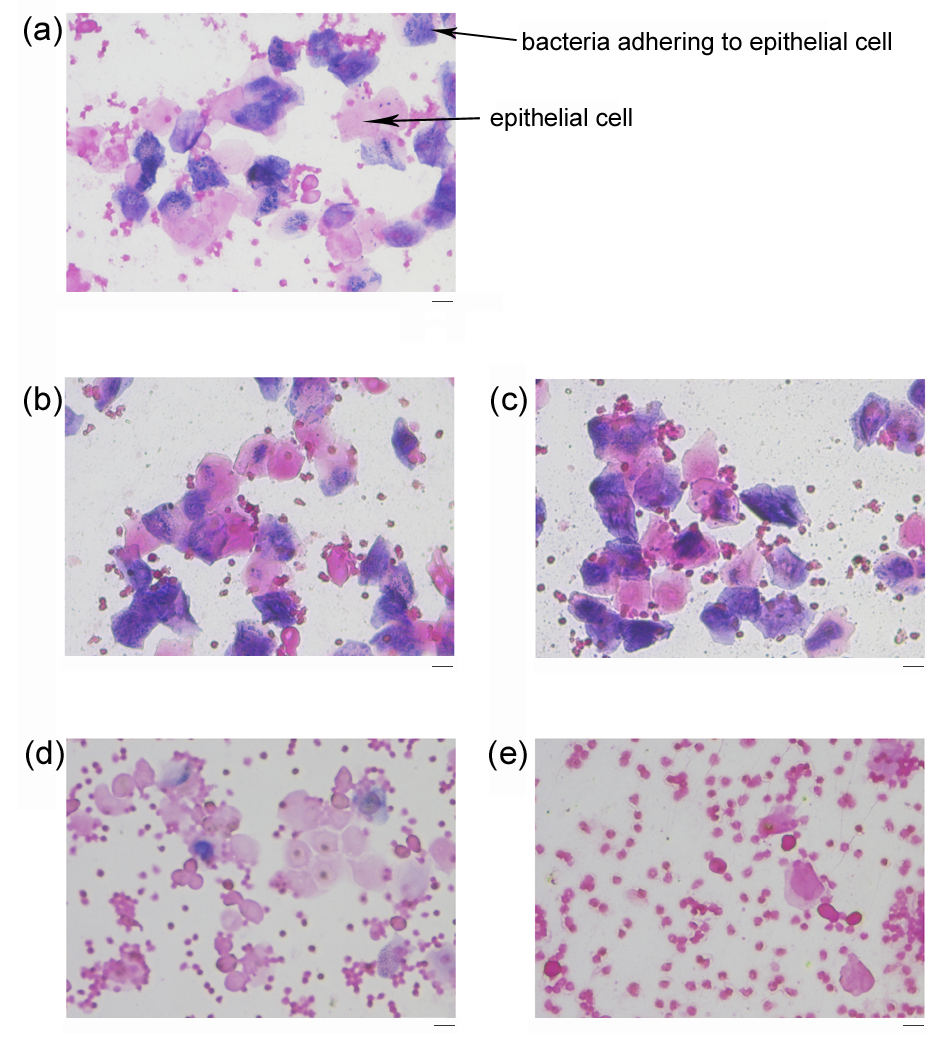

Supplement: Figure S2 — Bacteriostatic activity at different concentrations of minocycline gels. Vaginal smears were prepared from vagina CVL precipitants, and then gram stained and examined under microscopy at a 400× magnification. (a) represented the vagina smear from placebo treated group, (b), (c), (d) and (e) represented vagina smears from groups treated with 5 μg/ml, 50 μg/ml, 500 μg/ml and 5000 μg/ml minocycline gels respectively. Scale bar: 20 μm. (TIF) [file pone.0043211.s002.tif]
