# Supplementary material for: Minocycline Down-Regulates Topical Mucosal Inflammation during the Application of Microbicide Candidates
Source: PLoS One. 2012 Aug 14;7(8):e43211. doi: 10.1371/journal.pone.0043211 (PMC3419165; doi:10.1371/journal.pone.0043211)
Supplement: Table S1 — MIC of minocycline against bacteria isolates. The experiment was repeated for three times and data were represented as means ± SD. All bacteria strains were isolated from mice vagina and identified by GC-FAME analyses for their species notation with an instrument produced by Agilent Tech. (DOC) [file pone.0043211.s003.doc]

Table S1. MIC of minocycline against bacteria isolates.

| **Bacteria isolates** | **Gram stain** | **Value of MIC (μg/ml)^#^** |
| --- | --- | --- |
| *Pediococcus-pentosaceus* | positive | 0.8±0.12 |
| *Acidovorax-avenae-citrulli* | negative | 0.16±0.08 |
| *Stenotrophomonas-maltophilia* | negative | 0.8±0.07 |
| *Acidovorax-facilis* | negative | 0.16±0.21 |
| *Acinetobacter-lwoffii* | negative | 0.16±0.35 |
| *lactobacillus* | positive | 0.8±0.13 |

# The experiment was repeated for three times and data were represented as means ± SD. All bacteria strains were isolated from mice vagina and identified by GC-FAME analyses for their species notation with an instrument produced by Agilent Tech.
